# Linear Search by a Pair of Distinct-Speed Robots

**DOI:** 10.1007/s00453-018-0447-0

**Published:** 2018-05-07

**Authors:** Evangelos Bampas, Jurek Czyzowicz, Leszek Gąsieniec, David Ilcinkas, Ralf Klasing, Tomasz Kociumaka, Dominik Pająk

**Affiliations:** 1 LIS, CNRS and Aix-Marseille University, Marseille, France; 2 Département d’informatique, Université du Québec en Outaouais, Gatineau, Canada; 3 Department of Computer Science, University of Liverpool, Liverpool, UK; 4 LaBRI, CNRS, Univ. Bordeaux, Talence, France; 5 Institute of Informatics, University of Warsaw, Warsaw, Poland; 6 CSAIL, Massachusetts Institute of Technology, Cambridge, USA

**Keywords:** Linear search, Mobile robots, Group search, Different speeds

## Abstract

Two mobile robots are initially placed at the same point on an infinite line. Each robot may move on the line in either direction not exceeding its maximal speed. The robots need to find a stationary target placed at an unknown location on the line. The search is completed when both robots arrive at the target point. The target is discovered at the moment when either robot arrives at its position. The robot knowing the placement of the target may communicate it to the other robot. We look for the algorithm with the shortest possible search time (i.e. the worst-case time at which both robots meet at the target) measured as a function of the target distance from the origin (i.e. the time required to travel directly from the starting point to the target at unit velocity). We consider two standard models of communication between the robots, namely *wireless communication* and *communication by meeting*. In the case of communication by meeting, a robot learns about the target while sharing the same location with a robot possessing this knowledge. We propose here an optimal search strategy for two robots including the respective lower bound argument, for the full spectrum of their maximal speeds. This extends the main result of Chrobak et al. (in: Italiano, Margaria-Steffen, Pokorný, Quisquater, Wattenhofer (eds) Current trends in theory and practice of computer science, SOFSEM, 2015) referring to the exact complexity of the problem for the case when the speed of the slower robot is at least one third of the faster one. In the wireless communication model, a message sent by one robot is instantly received by the other robot, regardless of their current positions on the line. For this model, we design a strategy which is optimal whenever the faster robot is at most $\sqrt{17}+4\approx 8.123$ times faster than the slower one. We also prove that otherwise the wireless communication offers no advantage over communication by meeting.

## Introduction

Searching is a well-studied problem in which mobile robots need to find a specific target placed at some a priori unknown location. In some cases, a team of robots is involved, trying to coordinate their efforts in order to minimize the time. The complexity of the multi-robot searching is usually defined as the time when the first searcher arrives at the target position whose location is controlled by an adversary.

In distributed computing, one of the central problems is *rendezvous* when two mobile robots collaborate in order to meet in the smallest possible time. The efficiency of the rendezvous strategy is expressed as the time when the last involved robot reaches the meeting point, and the meeting point is arbitrary, i.e., the robots may choose the most convenient one.

In the *linear search* problem studied in the present paper, a pair of robots has to meet at an unknown fixed target point of the environment and the time complexity of the process is determined by the arrival of the second robot. More specifically, we consider two mobile robots placed at the origin of an infinite line. Each robot has its maximal speed that it cannot exceed while moving in either direction along the line. There is a stationary target, placed at an unknown point of the line, that a robot discovers when arriving at its placement. The robot which possesses the knowledge of the target position may communicate it to the other robot. We consider two communication models of the robots: *communication by meeting* when the robots can exchange information only while being located at the same position, and *wireless communication* when the robot finding the target may instantaneously inform the other robot of its position. We want to schedule the movement of both robots so that eventually each of them arrives at the target location. The cost of the schedule is the first time when both robots are present at the target position. We express it as a function of the distance between the target and the origin.

### Previous Work

The *linear search* problem for a single robot was introduced by Beck [7] and Bellman [8]. (The original problem of [7, 8] involves a probability distribution of placements of the target that the robot knows.) They proposed an optimal online algorithm with competitive ratio 9 (i.e., the worst-case ratio of its cost with respect to the offline cost). A variant of this question is the *cow-path problem* in [3], in which the searcher has more than two directions to follow. The original problem was also extended to searching in the plane [4], and numerous other variations [12, 15, 20, 22, 28, 29, 31, 32, 34]. Bose et al. [11] recently studied a variant of these problems where upper and lower bounds on the distance to the target are given. On a line, without this information several observations and partial results hint to the fact that the competitive ratio 9 cannot be improved even if the search is performed by a team of same-speed robots communicating by meeting if all robots have to reach the target [13]; see also [5]. Surprisingly, the same search time can still be achieved by distinct-speed robots if the slowest robot is at most 3 times slower than the fastest one [13].

### Our Results

In this paper, we consider the linear search problem for two robots equipped with distinct maximal speeds. For the convenience of presentation we scale their speeds so that the speed of the faster robot is 1 and the slower one is $0 < v \le 1$.

In the model with communication by meeting, we propose an optimal strategy for any value of *v*. In particular, our strategy works in time $\tfrac{1+3v}{v-v^2}d,$ for any $v \le \tfrac{1}{3}$  for the target being placed at unknown distance *d* from the origin, which yields a competitive ratio $\frac{1+3v}{1-v}$. The remaining part of the spectrum has been partially covered in [13] where the authors provide an argument for the lower bound $9d-o(d)$ when the robots share the maximal speed 1, under certain conditions on the algorithm, and they show that this bound can be met from above when the slower robot’s maximal speed is at least $\frac{1}{3}.$ We complement these results by providing the full formal proof of the lower bound $9d-o(d)$ for any set of robots with maximal speed at most 1. In the model with wireless communication, we design a simple strategy achieving search time $\frac{2+v+\sqrt{v^2 + 8v}}{2v}d$ and competitive ratio $\frac{2+v+\sqrt{v^2 + 8v}}{2}$. This algorithm for wireless communication outperforms the optimal strategy for communication by meeting for $v > \sqrt{17}-4 \approx 0.123$, which shows that the feature of wireless communication is useful in this range of parameters. Interestingly, this threshold is not an artifact of the particular algorithms we designed. We prove that for any *v* the optimum competitive ratio in the wireless communication model is achieved either by our strategy for wireless communication, or by the trivial adaptation of the optimal strategy for communication by meeting. Hence, for $v\le \sqrt{17}-4$, the wireless communication gives no advantage over communication by meeting.

### Related Work

Numerous papers have been written on the *searching problem*, studying diverse models involving stationary or mobile targets, graph or geometric terrain, known or unknown environment, one or many searchers, etc. (cf. [1, 2, 4, 26, 35]). Depending on the setting, the problem is known under the name of treasure hunting, pursuit-evasion, cops and robbers, fugitive search games, etc. Sometimes the searching robot is not looking for an individual target point, attempting rather to evacuate being lost in an unknown environment or determine its position within a known map (e.g. [18, 24]). Several of these research papers offer exciting challenges of combinatorial or algorithmic nature (see [26]). In most papers studying algorithmic issues, the objective is either to determine the feasibility of the search, (i.e., whether the search will succeed under all adversarial choices) or to minimize its cost represented by the search time, assuming some given speeds of searchers (and perhaps evaders).

Many searching algorithms are studied in the *online* setting (cf. [30]), where the information about the environment is acquired as the search progresses. The performance of an online algorithm is measured by its *competitive ratio*, i.e., the worst-case ratio of its cost with respect to the offline cost, which is the search time of the optimal algorithm with full a priori knowledge of the environment and the target placement. Many search problems, especially for geometric environments, are analyzed from this perspective, in particular when the cost of the offline solution is just the distance to the target; see [4, 13, 25, 30].

Most of the papers study the searching problem for a *single* robot. As a single searching robot usually cannot fully explore and map an arbitrary unknown graph (unless e.g., by leaving pebbles at some nodes; see [9]), a second searching robot is often necessary (and sufficient) in order to make the task feasible (cf. [10]). However, optimization of the search by the use of multiple robots often involves coordination issues, where the searchers need to communicate in order to synchronize their efforts and adequately split the entire task into portions assigned to individual robots (cf. [13, 23, 25, 27]). As this objective is often not easy to achieve, some multi-robot search problems turn out to be NP-hard (e.g., see [27]).

In previous research on the searching problem usually robots traveling at the *same* speed were considered (cf. [13, 14, 17, 18, 21]). For other problems considering robots with distinct speeds (e.g., the patrolling problem studied in [16, 19, 33]), only partial results were obtained. Optimal patrolling using more than two robots on a ring [19], or more than three robots on a segment [33], is unknown in general and all intuitive solutions have been proved sub-optimal for some configurations of the speeds of the robots. Another example is the long-standing *lonely runner* conjecture [37], concerning *k* entities moving with constant speeds around a circular track of unit-length. If the speeds are pairwise different, the conjecture states that at some moment all runners are located equidistantly on the cycle. The conjecture is open in general, having been verified for up to 7 runners [6].

A closely related problem, the *rendezvous problem*, has been central to distributed computing for many years. It was studied in various settings (cf. [36]), but even for environments as simple as a line or a ring, optimal solutions are not always known. Feasibility of the rendezvous problem is often determined by a symmetry breaking process, which must prevent the robots from falling into an infinite pattern avoiding the meeting. Searching and rendezvous may be viewed as problems with opposite objectives. Searching is a game between a searcher, who tries to find the target as fast as possible and the adversary, who knows the searching strategy and attempts to maximize the search time by its choice of the environment parameters, target placement (or its escape route), etc. Hence in searching, the two players have contradictory goals. In rendezvous the two players collaborate, trying to quickly find one another (see [2]). Contrary to the searching problem, the rendezvous destination is not given in advance but it may be decided by the robots.

## Preliminaries

For any algorithm $\mathcal {A}$, we denote by $t(\mathcal {A},p)$ the *search time* of algorithm $\mathcal {A}$ if the target is located at point *p*. In other words, this is the time at which all robots meet at the target *p*. As it is standard in the literature, we assume that the target is at a distance of at least 1 away from the origin.

In the offline setting, if the robots know the target, the search time is clearly $\frac{1}{v}|p|$, where *v* is the speed of the slowest robot. We use the *competitive ratio*$\mathsf {CR}(\mathcal {A})$ of algorithm $\mathcal {A}$, equal to

$$\begin{aligned} \mathsf {CR}(\mathcal {A}) = \sup _{|p|\ge 1}\tfrac{v \cdot t(\mathcal {A},p)}{|p|}, \end{aligned}$$

as the main efficiency measure of the algorithms. In what concerns lower bounds, we actually prove stronger lower bounds for the quantity

$$\begin{aligned} \tau (\mathcal {A})=\limsup _{|p|\rightarrow \infty }\frac{t(\mathcal {A},p)}{|p|}. \end{aligned}$$

They imply lower bounds for the competitive ratio due to $\mathsf {CR}(\mathcal {A})\ge v\cdot \tau (\mathcal {A})$.

Having fixed an algorithm $\mathcal {A}$ for a set $\mathcal {R}$ of robots, each robot $\varGamma \in \mathcal {R}$ follows a fixed trajectory as long as it is unaware of the location of the target. We use $\varGamma (t)$ to denote the position of robot $\varGamma$ at time *t* provided that the target location is not known to the robot. Our lower bounds rely on the analysis of the *progress speeds*$\limsup _{t\rightarrow \infty } \frac{|\varGamma (t)|}{t}$. The largest of these values over $\varGamma \in \mathcal {R}$ is called the *overall progress speed*. For each point *p*, the time $T(p) = \min \{t : \exists {\varGamma \in \mathcal {R}} \quad \varGamma (t) = p\}$ is called the *discovery time* of *p* (it is the first moment when any robot visits *p*). For each time *t*, we denote $D(t)=\{p : T(p)\le t\}$ the segment of points discovered until time *t*. We call the value $\liminf _{t\rightarrow \infty } \frac{|D(t)|}{t}$ the *discovery speed*.

Our results are primarily designed for a set $\mathcal {R}$ of two robots, denoted *R* and *r*. Their speed limits are 1 and *v* ($0 < v \le 1$), respectively.

## Communication by Meeting

In this model, once a robot finds the target, it must walk to meet the other robot, and then the robots travel to the target. Naturally, the schedule consists of three phases: the *exploration phase* while the target is unknown, the *pursuit phase* where the informed robot chases after the other one in order to tell it about the target, and the *target phase* when both robots walk to the target location. Recall that for robots with equal speeds, one of the possible (optimal) solutions consists in all the robots following together the same cow-path trajectory [5, 13], thus the pursuit and target phases may be nonexistent.

### The Upper Bound

Let us first recall the structure of the cow-path trajectory. A robot visits, for subsequent integers $k\in \mathbb {N}$, the points $p_k := (-\,2)^k$ on alternating sides of the origin, traveling at full speed between consecutive points $p_k$. In this strategy, the robot discovers new locations after it passes $p_k$ on the way from $p_{k+1}$ to $p_{k+2}$. This happens from time $t_k := |p_k|+2\sum _{j=0}^{k+1} |p_j| = 9\cdot 2^k - 2= 9 |p_k| - 2$ to $t'_{k+2}:=|p_{k+2}|+2\sum _{j=0}^{k+1} |p_j| = 12\cdot 2^k - 2 = 3|p_{k+2}| - 2$. Consequently, the search time is bounded from above by 9|*p*|.

**Fig. 1:**
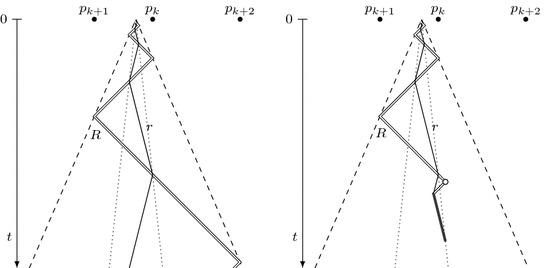
Illustration of algorithm $\mathcal {A^*}$ before target detection (left), and when the target has been located (right). The horizontal axis represents the line searched and the vertical axis represents the time. The empty circle denotes the target discovery. Double and single solid lines represent the trajectories of the faster and the slower robot, respectively. Dashed lines correspond to the overall progress speed and dotted lines to the search time

As observed by Chrobak et al. [13], this strategy generalizes to a collection of two robots with speed limits 1 and $\frac{1}{3}$. Both robots follow the cow-path trajectory at their maximal speed, which means that they meet in $p_k$ at time $t_k = 3t'_k$. When the faster robot *R* discovers the target at a point *p* between $p_k$ and $p_{k+2}$, it pursues the slower robot *r* and brings it to the target, which turns out to be feasible within time 9|*p*|; see Fig. 1.

**Table:** 

**Algorithm**$\mathcal {A^*}$ [for two robots with communication by meeting]	
1. Until the target is located, both robots visit, in order of increasing *k*, the points $p_k=(-c)^k$ for all $k\in \mathbb {N}$, where $c=\frac{1+\tilde{v}}{2\tilde{v}}$ and $\tilde{v}=\min (v,\frac{1}{3})$. Robot *R* moves with speed 1 between consecutive points, and robot *r* with speed $\tilde{v}$. Robot *R* starts its trajectory at time 0, whereas robot *r* initially waits at the origin for 4 time units.	
2. When *R* finds the target, it moves with speed 1 to meet and notify *r*.	
3. After the meeting, the robots move together to the target at speed $\tilde{v}$.	

We extend this strategy to allow $v<\frac{1}{3}$ as the speed limit of the slower robot *r*. We insist on the two robots meeting in points $p_k$ at times $t_k$ for adjusted values $p_k$ and $t_k$. The smaller speed *v* of *r* allows *R* to travel further before going back to $p_k$. More formally, we increase the ratio $|p_{k+1}|/|p_k|$ and instead of taking $p_k=(-\,2)^k$, we set $p_k=(-\,c)^k$ for some $c>2$. We still make both robots visit consecutive points $p_k$ at their full speeds, and we choose *c* so that they meet in $p_k$ while *r* is there for the first time and *R* for the second time. A condition inductively forcing the meeting at $p_{k}$ to be followed by a meeting in $p_{k+1}$ can be expressed as $\frac{1}{v}|p_{k+1}-p_{k}| = t_{k+1}-t_k= |p_{k+1}-p_{k+2}|+|p_{k+2}-p_{k}|$, i.e., $\frac{1}{v} (c+1)=2c^{2}+c-1$. This gives $c=\frac{1+v}{2v}$, which we use for our algorithm $\mathcal {A^*}$. The meeting at $p_0$ is guaranteed by delaying *r* for 4 time units: Indeed, *r* arrives at $p_0$ for the first time at time $t_0=4+\frac{1}{v}$ and *R* arrives at $p_0$ for the second time at time $3+2c$, which is equal to $t_0$ for our choice of *c*.

The following theorem bounds the search time by robots using this strategy.

#### Proposition 3.1

For the algorithm $\mathcal {A^*}$ and every point $p\in \mathbb {R}$ with $|p|\ge 1$, we have:

1 $$\begin{aligned} t(\mathcal {A^*},p)< \tfrac{1+3v}{v-v^2}|p|&\quad \text { if }\ v\le \tfrac{1}{3}, \end{aligned}$$

2 $$\begin{aligned} t(\mathcal {A^*},p)< 9|p|&\quad \text { if }\ \tfrac{1}{3} \le v \le 1. \end{aligned}$$

#### Proof

First, let us show (1). Assuming that there exists *k* so that the target *p* is located between $p_k$ (exclusive) and $p_{k+2}$ (inclusive), the meeting time in $p_k$ is

$$\begin{aligned} t_k = 4+ \tfrac{1}{v} \left( |p_k|+2\sum _{j=0}^{k-1}|p_j|\right) = 4 + \tfrac{1}{v}c^k\tfrac{c+1}{c-1} - \tfrac{2}{v(c-1)} =c^k\tfrac{1+3v}{v-v^2} - 4\tfrac{v}{1-v}. \end{aligned}$$

Suppose that $|p-p_k|=\delta$. After meeting *r* in $p_k$, robot *R* needs time $\delta$ to discover the target. At that time, the distance between the robots is $\delta (1+v)$ since they are going in opposite directions with their maximal speeds until time $t_k+\delta$. Then, the faster robot pursues the slower one. With the speed difference of $1-v$ this takes $\frac{\delta (1+v)}{1-v}$ units of time. Next, the robots go back to the target at speed *v* which requires time $\frac{\delta (1+v)}{v-v^2}$, i.e., $\frac{1}{v}$ times more than the pursuit. In total, the time between $t_k$ and the moment when both robots reach the target is

$$\begin{aligned} \delta + \frac{\delta (1+v)}{1-v}+ \frac{\delta (1+v)}{v-v^2}=\delta \frac{v-v^2+v+v^2+1+v}{v-v^2}=\delta \frac{1+3v}{v-v^2}. \end{aligned}$$

Since $t_k < |p_k|\tfrac{1+3v}{v-v^2}$, the total search time is $t(\mathcal {A^*},p) < (|p_k|+\delta )\tfrac{1+3v}{v-v^2}=|p|\tfrac{1+3v}{v-v^2}$, as claimed.

If no such *k* exists, then we must have $p=1$ or $-c\le p\le -1$. It is easy to verify that $t(\mathcal {A^*},1)=2+\tfrac{1}{v}<\tfrac{1+3v}{v-v^2}$. Moreover, if $-c\le p\le -1$, *R* discovers the target and comes back to the origin at time $2+2|p|$. At the same time, *r* is at point $2(|p|-1)v$ and it is moving away from *R*. Therefore, it takes an additional $\tfrac{2(|p|-1)v}{1-v}(1+\tfrac{1}{v}) + \tfrac{|p|}{v}$ time before both robots evacuate. Overall, in this case, $t(\mathcal {A^*},p)=2(|p|+1)+\tfrac{2(|p|-1)v}{1-v}(1+\tfrac{1}{v}) + \tfrac{|p|}{v}=\frac{1+3v}{v-v^2}|p|-\frac{4v}{1-v}$.

To show (2), we simply observe that, for $v= \frac{1}{3}$, we have $\frac{1+3v}{v-v^2} = 9$. Note that for $v > \frac{1}{3}$, the searcher moving at velocity $\frac{1}{3}$ could increase its speed to *v*, but no additional gain in efficiency is possible (see the lower bounds in [5, 13] and in Sect. 3.2). $\square$

#### Corollary 3.2

For the algorithm $\mathcal {A^*}$, $\mathsf {CR}(\mathcal {A^*})\le \tfrac{1+3v}{1-v}$ if $v\le \tfrac{1}{3}$, and $\mathsf {CR}(\mathcal {A^*})\le 9v$ if $v\ge \tfrac{1}{3}$.

### The Lower Bound

Below, we show that the strategy from Sect. 3.1 achieves the best possible competitive ratio. We first present a sketch of the arguments behind the intermediate results used for this aim. In fact, some of these lemmas are stated so that they work in more general settings.

**Fig. 2:**
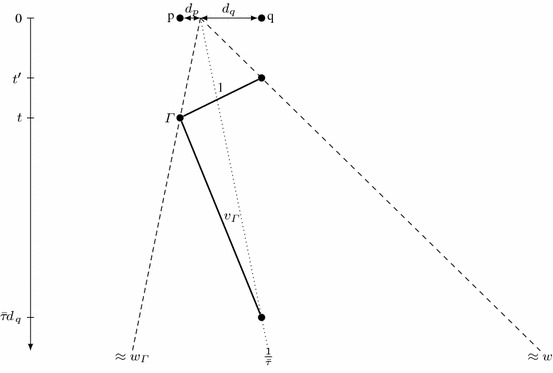
Illustration of notions used in the proof of Lemma 3.3. Rays starting from the origin as well as thick lines representing constraints are all annotated with the corresponding speeds. Here, robot $\varGamma$, while in *p* at time *t*, must know that the target is not in *q*, or it must be able to reach *q* before the deadline

We first study a hypothetical algorithm $\mathcal {A}$ for a collection $\mathcal {R}$ of any number of robots, each with maximum speed not exceeding 1. In Lemma 3.3, we analyze it from the perspective of a robot $\varGamma \in \mathcal {R}$ with maximum speed $v_{\varGamma }$ and progress speed $w_{\varGamma }$ (not exceeding the overall progress speed *w*). By definition of the progress speed, the robot $\varGamma$ sometimes visits points $p=\varGamma (t)$ with $|p|\approx t\cdot w_{\varGamma }$. We fix such a point *p* and choose a point *q* on the opposite side of the origin, with deadline $t(\mathcal {A},q)\approx \tau (A)\cdot |q|$ sufficiently early that the robot $\varGamma$, starting in *p* at time *t*, cannot reach *q* before the deadline. We deduce that the robot $\varGamma$ must already know that the target is not located at *q*. Such an information, conveyed by one or more robots, can be transferred with maximum speed 1, which yields an upper bound on the discovery time *T*(*q*) of the point *q*. On the other hand, the overall progress speed implies a lower bound of approximately $\frac{|q|}{w}$ on *T*(*q*); see Fig. 2. Combining these two constraints, after some calculations we obtain the inequality $\tau (\mathcal {A})\ge \frac{v_{\varGamma }+w_\varGamma + w + v_{\varGamma }w}{v_{\varGamma }w(1-w_\varGamma )}$.

**Fig. 3:**
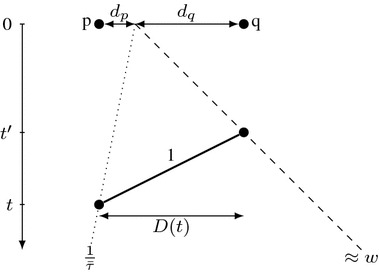
Illustration of notions used in the proof of Lemma 3.7

We examine the consequences of this result in Corollaries 3.4, 3.5 and 3.6. Setting $\varGamma$ as the robot with progress speed $w_\varGamma =w$, we derive $\tau (\mathcal {A})\ge \frac{1+3w}{w-w^2}\ge 9$ (Corollary 3.4). This immediately shows that any algorithm $\mathcal {A}$ with $\tau (\mathcal {A})<\tau (\mathcal {A^*})$ must have its overall progress speed *w* strictly smaller than the overall progress speed $\frac{1-v}{1+3v}$ of $\mathcal {A^*}$ (Corollary 3.5). On the other hand, setting $\varGamma$ as the slowest robot *r* with maximum speed *v*, we conclude that unless $\tau (\mathcal {A})\ge \tau (\mathcal {A^*})$, the progress speed $w_r$ of *r* is also strictly smaller than the progress speed of the slower robot *r* in $\mathcal {A^*}$, equal to $\frac{1}{\tau (\mathcal {A^*})}$. Consequently, the robot *r* may only discover points at bounded distance from the origin (Corollary 3.6). Thus, in the final part of the proof we analyze a hypothetical two-robot algorithm $\mathcal {A}$ in which only the faster robot *R* discovers sufficiently far points. (Note that $\mathcal {A^*}$ satisfies this condition.) Since the slower robot *r* does not participate in the exploration, the discovery speed depends on the trajectory of the faster robot *R* only. This lets us relate the discovery speed $v_d$ to the progress speed *w* (Lemma 3.7). By Corollary 3.4, there are points *p* with discovery time *T*(*p*) exceeding approximately $(\frac{1+3w}{w-w^2})^{-1} |p|$. To bound the length of *D*(*T*(*p*)), we observe that the other endpoint *q* of this segment has its discovery time *T*(*q*) bounded from below due to the progress speed *w* and from above due to the maximum speed 1; see Fig. 3. After some calculations we achieve $v_d \le \frac{2w}{1+3w}$.

**Fig. 4:**
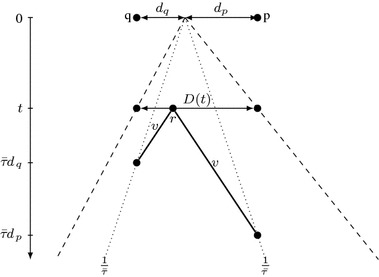
Illustration of notions used in the proof of Lemma 3.8. The slowest robot *r* must be able to reach *p* by $\bar{\tau }d_p$ and *q* by $\bar{\tau }d_q$

Finally, we prove a lower bound on the discovery speed $v_d$ in terms of $\tau (\mathcal {A})$ and the speed limit *v* of the slower robot *r* (Lemma 3.8). For this, we just note that at any time *t*, the robot *r* must take into account that the target is located arbitrarily close to either endpoint of the segment *D*(*t*) (see Fig. 4), which yields $v_d \ge \frac{2v}{v\tau (\mathcal {A})-1}$. We combine the two bounds on discovery speed $v_d$ with the upper bound on the overall progress speed *w* to prove that if the set of points discovered by *r* is bounded, then $\tau (\mathcal {A})\ge \tau (\mathcal {A^*})$. Interestingly, our proof remains valid in the wireless communication model; we exploit this fact in Sect. 4.2. We conclude with the lower bound on $\tau (\mathcal {A})$ in Proposition 3.10. In Theorem 3.11, we deduce that the algorithm $\mathcal {A^*}$ has optimal competitive ratio.

We will now formally prove all these results. First, let us relate the search time and the progress speeds in an algorithm $\mathcal {A}$ for any collection $\mathcal {R}$ of robots.

#### Lemma 3.3

Let $\mathcal {A}$ be a line search algorithm with overall progress speed *w* for a collection $\mathcal {R}$ of robots with speeds not exceeding 1, communicating by meeting. If there is a robot $\varGamma \in \mathcal {R}$ with speed limit $v_{\varGamma }$ and progress speed $w_{\varGamma }$, then $\tau (\mathcal {A})$ is unbounded provided that $w=0$ or $w_{\varGamma }=1$, and $\tau (\mathcal {A})\ge \frac{v_{\varGamma }+w_{\varGamma }+w+v_{\varGamma }w}{v_{\varGamma }w(1-w_{\varGamma })}$ otherwise.

#### Proof

Let us choose $\bar{\tau },\varepsilon \in \mathbb {R}$ so that $\bar{\tau }> \tau (\mathcal {A})$ and $\varepsilon > 0$. Then, there exists $d_0>0$ such that $\frac{1}{\bar{\tau }}t(\mathcal {A},p)< |p| < (w+\varepsilon ) T(p)$ for $|p|\ge d_0$. Also, there are arbitrarily large times *t* such that $\frac{|\varGamma (t)|}{t}\ge w_{\varGamma }-\varepsilon$; we fix one with $t\ge \bar{\tau }d_0$.

Let $p=\varGamma (t)$ and $d_p = |p|$. Also, consider a point *q* at distance $d_q=\frac{v_{\varGamma }t+d_p}{v_{\varGamma }\bar{\tau }-1}$ from the origin, opposite to *p*; see Fig. 2. Note that $d_q\ge \frac{t}{\bar{\tau }}\ge d_0$, so $\tfrac{1}{\bar{\tau }}t(\mathcal {A},q)< d_q < (w+\varepsilon )T(q)$.

Suppose that the robot $\varGamma$ at time *t* cannot exclude the possibility that the target is located at *q*. Then, it must be able to reach *q* by the deadline, at $t(\mathcal {A},q)<\bar{\tau }d_q$. Since $\varGamma (t)=p$ and the robot $\varGamma$ cannot exceed the speed limit of $v_{\varGamma }$, we conclude that $t + \frac{1}{v_{\varGamma }}(d_p+d_q) < \bar{\tau }d_q$. However, the distance $d_q$ is defined so that $t + \frac{1}{v_{\varGamma }}(d_p+d_q) = \bar{\tau }d_q$, a contradiction.

Consequently, the robot $\varGamma$ must already know at time *t* that the target is not at point *q*. The robots can only communicate by meeting and their speeds are limited by 1, so this information needs at least $d_q+d_p$ time to be transferred from *q* to *p*. In other words, some robot $\varGamma '$ must have visited *q* at time $T(q)\le t-d_p-d_q$.

Combined with $d_q < (w+\varepsilon )T(q)$, this implies $d_q < (w+\varepsilon )(t-d_p-d_q)$, i.e., $d_q < \frac{(w+\varepsilon )(t-d_p)}{1+w+\varepsilon }$. By definition of $d_q$, this further yields

$$\begin{aligned} \tfrac{v_{\varGamma }t+d_p}{v_{\varGamma }\bar{\tau }- 1}&<\tfrac{(w+\varepsilon )(t-d_p)}{1+w+\varepsilon },\\ (v_{\varGamma }t+d_p)(1+w+\varepsilon )&<(w+\varepsilon )(t-d_p)(v_{\varGamma }\bar{\tau }- 1),\\ d_p(1+w+\varepsilon + (w+\varepsilon )(v_{\varGamma }\bar{\tau }- 1))&<t((w+\varepsilon )(v_{\varGamma }\bar{\tau }-1)-v_{\varGamma }(1+w+\varepsilon )),\\ d_p(1+ (w+\varepsilon )v_{\varGamma }\bar{\tau })&<t((w+\varepsilon )(v_{\varGamma }\bar{\tau }-1-v_{\varGamma })-v_{\varGamma }),\\ \tfrac{d_p}{t}&< \tfrac{(w+\varepsilon )(v_{\varGamma }\bar{\tau }-1-v_{\varGamma })-v_{\varGamma }}{(w+\varepsilon )v_{\varGamma }\bar{\tau }+1}. \end{aligned}$$

However, recall that time *t* was chosen so that $d_p \ge (w_{\varGamma }-\varepsilon )t$. Therefore,

$$\begin{aligned} w_{\varGamma }-\varepsilon < \tfrac{(w+\varepsilon )(v_{\varGamma }\bar{\tau }-1-v_{\varGamma })-v_{\varGamma }}{(w+\varepsilon )v_{\varGamma }\bar{\tau }+1}. \end{aligned}$$

As $\varepsilon >0$ can be chosen arbitrarily close to 0, we conclude that

$$\begin{aligned} w_\varGamma&\le \tfrac{w(v_{\varGamma }\bar{\tau }-1-v_{\varGamma })-v_{\varGamma }}{wv_{\varGamma }\bar{\tau }+ 1},\\ w_\varGamma wv_{\varGamma }\bar{\tau }+ w_\varGamma&\le w(v_{\varGamma }\bar{\tau }-1-v_{\varGamma })-v_{\varGamma },\\ w_\varGamma +w+v_{\varGamma }w+v_{\varGamma }&\le \bar{\tau }v_{\varGamma }w(1-w_\varGamma ). \end{aligned}$$

If $w=0$ or $w_{\varGamma }=1$, this is a contradiction: $0 = wv_{\varGamma }\bar{\tau }(1-w_{\varGamma }) \ge v_{\varGamma }+w_{\varGamma }+w+v_{\varGamma }w\ge v_{\varGamma } > 0$. Hence, $\tau (\mathcal {A})$ cannot be bounded from above. Otherwise, $wv_{\varGamma }(1-w_{\varGamma })>0$, so $\bar{\tau }\ge \frac{v_{\varGamma }+w_{\varGamma }+w+v_{\varGamma }w}{v_{\varGamma }w(1-w_{\varGamma })}$. Since $\bar{\tau }$ can be chosen arbitrarily close to $\tau (\mathcal {A})$, we conclude that $\tau (\mathcal {A})\ge \frac{v_{\varGamma }+w_{\varGamma }+w+v_{\varGamma }w}{v_{\varGamma }w(1-w_{\varGamma })}$. $\square$

The following immediate corollary gives a complete proof of the lower bound 9 in the general case; it also proves the optimality of $\mathcal {A^*}$ for $v \ge \frac{1}{3}$. (Recall the partial arguments of the lower bound of 9 in [13]; see also [5].)

#### Corollary 3.4

For any algorithm $\mathcal {A}$ with overall progress speed *w* and any collection $\mathcal {R}$ of robots with speeds not exceeding 1 and communicating by meeting, we have $\tau (\mathcal {A})\ge \frac{1+3w}{w-w^2}\ge 9$.

#### Proof

We apply Lemma 3.3 for the robot $\varGamma$ with progress speed *w* and speed $v_{\varGamma } \le 1$. We obtain

$$\begin{aligned} \tau (\mathcal {A})\ge \tfrac{v_{\varGamma }+2w+v_{\varGamma }w}{v_{\varGamma }(w-w^2)}=\tfrac{1+\tfrac{2w}{v_{\varGamma }}+w}{w-w^2}\ge \tfrac{1+3w}{w-w^2}. \end{aligned}$$

Finally, we observe that

$$\begin{aligned} \tfrac{1+3w}{w-w^2}-9 = \tfrac{1+3w-9w+9w^2}{w-w^2}=\tfrac{(1-3w)^2}{w-w^2}\ge 0, \end{aligned}$$

so $$\tau (\mathcal {A})\ge 9$$. $$\square $$

Another straightforward corollary proves the optimality of $\mathcal {A^*}$ provided that the progress speed *w* is sufficiently large.

#### Corollary 3.5

Let $\mathcal {A}$ be a line search algorithm for any collection $\mathcal {R}$ of robots with speeds not exceeding 1 and communicating by meeting. If the overall progress speed satisfies $w\ge \frac{1-v}{1+3v}$, then $\tau (\mathcal {A})\ge \tau (\mathcal {A^*})$.

#### Proof

Observe that the function $f(v)=\frac{1+3v}{v-v^2}$ is decreasing for $0<v\le \frac{1}{3}$ and increasing for $\frac{1}{3} \le v < 1$. For $v\le \frac{1}{3}$, we have $w \ge \frac{1-v}{1+3v} > \frac{1-\frac{1}{3}}{1+1} = \frac{1}{3}$, so Corollary 3.4 yields

$$\begin{aligned} \tau (\mathcal {A}) \ge f(w)\ge f(\tfrac{1-v}{1+3v}) = \tfrac{1+3v}{v-v^2} \ge \tau (\mathcal {A^*}). \end{aligned}$$

On the other hand, for $v \ge \frac{1}{3}$, Corollary 3.4 implies

$$\begin{aligned} \tau (\mathcal {A}) \ge f(w)\ge f(\tfrac{1}{3}) = 9 \ge \tau (\mathcal {A^*}). \end{aligned}$$

In both cases we derived the claimed inequality. $\square$

Next, we conclude that in any algorithm beating $\mathcal {A^*}$, the slowest robot *r* cannot discover arbitrarily far points.

#### Corollary 3.6

Let $\mathcal {A}$ be a line search algorithm for a collection $\mathcal {R}$ of robots with speeds not exceeding 1 and communicating by meeting. If the set of points discovered by the slowest robot *r* with speed *v* is unbounded, then $\tau (\mathcal {A})\ge \tau (\mathcal {A^*})$.

#### Proof

For a proof by contradiction, suppose that $\tau (\mathcal {A})<\tau ^*$, where $\tau ^*= \tau (\mathcal {A^*})$. By Corollary 3.5, this yields a bound $w<\frac{1-v}{1+3v}\le \frac{1}{v\tau ^*}$ on the overall progress speed. Moreover, if *r* discovers arbitrarily far points, then its progress speed $w_r$ satisfies $w_r \ge \frac{1}{\tau (\mathcal {A})}>\frac{1}{\tau ^*}$. Hence, Lemma 3.3 applied for the slowest robot *r* yields

$$\begin{aligned} \tau ^*> \tau (\mathcal {A}) \ge \tfrac{v+w_r+w+vw}{vw(1-w_r)} \ge \frac{v+\tfrac{1}{\tau ^*} +\tfrac{1}{v\tau ^*}+\tfrac{v}{v\tau ^*}}{v\tfrac{1}{v\tau ^*}\left( 1-\tfrac{1}{\tau ^*}\right) }= \tau ^*\tfrac{v^2\tau ^*+ 2v+1}{v(\tau ^*-1)}. \end{aligned}$$

In other words,

$$\begin{aligned} v\tau ^*- v&> v^2\tau ^*+ 2v + 1,\\ \tau ^*&> \tfrac{1+3v}{v-v^2}. \end{aligned}$$

This contradiction concludes the proof. $\square$

Next, we aim at showing that $\tau (\mathcal {A})\ge \tau (\mathcal {A^*})$ provided that the set of points discovered by the slower robot *r* is bounded. Our proof does not rely on the communication by meeting model, so we state this result in the wireless communication model. We start with two bounds on the discovery speed and then deduce the claimed inequality with some calculations.

#### Lemma 3.7

Let $\mathcal {A}$ be a line search algorithm for a single robot with maximum speed 1, progress speed *w*, and discovery speed $v_d$. Then $v_d \le \tfrac{2w}{1+3w}.$

#### Proof

Let $\varepsilon >0$; then $|p|< (w+\varepsilon )T(p)$ for points *p* sufficiently far from the origin. Moreover, let $\bar{\tau }<\tfrac{1+3w}{w-w^2}$ and observe that, by Corollary 3.4, $\tau (\mathcal {A})>\bar{\tau }$; thus, there are arbitrarily far points *p* with $t(\mathcal {A},p)>\bar{\tau }|p|$.

Let us choose such a point *p* discovered at time $t:=T(p)=t(\mathcal {A},p)>\bar{\tau }|p|$, and let *q* be the furthest point on the opposite side of the origin, discovered at time $t':= T(q)<t$. Denote $d_p = |p|$ and $d_q = |q|$; see Fig. 3. The distance $d_q$ can be arbitrarily large provided that *p* is chosen sufficiently far; therefore, $d_q < (w+\varepsilon )t'$. Furthermore, the speed limit yields $t \ge t' + d_p+d_q$, so

$$\begin{aligned} d_q&< (w+\varepsilon )(t-d_p-d_q)\\ d_q(1+w+\varepsilon )&< (w+\varepsilon )(t-d_p)\\ d_q&< \tfrac{(w+\varepsilon )(t-d_p)}{1+w+\varepsilon } \end{aligned}$$

Now, observe that

$$\begin{aligned} \tfrac{|D(t)|}{t} = \tfrac{d_p+d_q}{t}< \tfrac{d_p+\tfrac{(w+\varepsilon )(t-d_p)}{1+w+\varepsilon }}{t}=\tfrac{d_p+(w+\varepsilon )t}{t(1+w+\varepsilon )}< \tfrac{1+\bar{\tau }(w+\varepsilon )}{\bar{\tau }(1+w+\varepsilon )}. \end{aligned}$$

Since *t* can be chosen arbitrarily large, we obtain $v_d \le \tfrac{1+\bar{\tau }(w+\varepsilon )}{\bar{\tau }(1+w+\varepsilon )}.$ Because $\varepsilon$ can be chosen arbitrarily close to 0 and $\bar{\tau }$ can be chosen arbitrarily close to $\tfrac{1+3w}{w-w^2}$, we furthermore conclude that

$$\begin{aligned} v_d \le \frac{1+\tfrac{1+3w}{w-w^2}w}{\tfrac{1+3w}{w-w^2}(1+w)}=\tfrac{w-w^2+w(1+3w)}{(1+3w)(1+w)}=\tfrac{2w+2w^2}{(1+3w)(1+w)} = \tfrac{2w}{1+3w}, \end{aligned}$$

as claimed. $$\square $$

#### Lemma 3.8

Let $\mathcal {A}$ be a line search algorithm for a collection $\mathcal {R}$ of robots, using *wireless communication* or *communication by meeting*. If $\mathcal {R}$ contains a robot *r* with speed *v*, then the discovery speed $v_d$ must satisfy $v_d \ge \tfrac{2v}{v\tau (\mathcal {A})-1}.$

#### Proof

Let $\bar{\tau }$ be a real number such that $\bar{\tau }> \tau (\mathcal {A})$; then $t(\mathcal {A},p)< \bar{\tau }|p|$ for points *p* sufficiently far from the origin.

Let *p* and *q* be furthest points on the opposite sides of the origin discovered at or before some time *t*. Moreover, let $d_p = |p|$ and $d_q = |q|$; see Fig. 4. Note that $d_p$ and $d_q$ are arbitrarily large provided that *t* is chosen sufficiently large. At time *t*, there are undiscovered points arbitrarily close to *p* and *q*. Hence, the robot *r* must be able to reach *p* before $\bar{\tau }d_p$ and *q* before $\bar{\tau }d_q$, i.e., $\bar{\tau }d_p \ge t + \frac{1}{v}|p-r(t)|$ and $\bar{\tau }d_q \ge t + \frac{1}{v}|q-r(t)|$. The point *r*(*t*) is located between *p* and *q*, so these two inequalities yield

$$\begin{aligned} \bar{\tau }(d_p+d_q)&\ge 2t + \tfrac{1}{v}\left( d_p+d_q\right) ,\\ (d_p+d_q)(v\bar{\tau }- 1)&\ge 2tv,\\ \tfrac{|D(t)|}{t}=\tfrac{d_p+d_q}{t}&\ge \tfrac{2v}{v\bar{\tau }-1}. \end{aligned}$$

This inequality holds for each sufficiently large *t*, so we have

$$\begin{aligned} v_d=\liminf _{t\rightarrow \infty } \tfrac{|D(t)|}{t} \ge \tfrac{2v}{v\bar{\tau }-1}. \end{aligned}$$

To derive the claimed bound on $v_d$, we note that $\bar{\tau }$ can be chosen arbitrarily close to $\tau (\mathcal {A})$. $\square$

#### Corollary 3.9

Let $\mathcal {A}$ be a line search algorithm for two robots *R* and *r* with speeds 1 and *v*, respectively, using *wireless communication* or *communication by meeting*. If the set of points discovered by *r* is bounded, then $\tau (\mathcal {A})\ge \tau (\mathcal {A^*})$.

#### Proof

For a proof by contradiction, suppose that $\tau (\mathcal {A}) < \tau (\mathcal {A^*})$. The trajectory of *R* can be interpreted as a line search algorithm $\mathcal {A}'$ for a collection $\mathcal {R}=\{R\}$ of one robot. Since the set of points discovered by *r* is bounded, the discovery speeds of $\mathcal {A}$ and $\mathcal {A}'$ are the same, say $v_d$. For the same reason, we have $\tau (\mathcal {A}')\le \tau (\mathcal {A})$, and, due to Corollary 3.5, $w < \frac{1-v}{1+3v}$. By Lemma 3.7, this yields

$$\begin{aligned} v_d \le \tfrac{2w}{1+3w} < \tfrac{2\frac{1-v}{1+3v}}{1+3\frac{1-v}{1+3v}}=\tfrac{2(1-v)}{1+3v+3(1-v)}=\tfrac{2(1-v)}{4}=\tfrac{1-v}{2}. \end{aligned}$$

On the other hand, Lemma 3.8 implies

$$\begin{aligned} v_d \ge \tfrac{2v}{v\tau (\mathcal {A})-1} > \tfrac{2v}{v\tau (\mathcal {A^*})-1}\ge \frac{2v}{v\tfrac{1+3v}{v-v^2}-1}=\tfrac{2v(1-v)}{1+3v-(1-v)}=\tfrac{1-v}{2}. \end{aligned}$$

This is a contradiction. $\square$

Corollaries 3.6 and 3.9 prove $\tau (\mathcal {A})\ge \tau (\mathcal {A^*})$ under complementary assumptions. Hence, we obtain the main result of this section.

#### Proposition 3.10

Any algorithm $\mathcal {A}$ in the *communication by meeting* model for two robots with speeds 1 and *v*, respectively, satisfies $\tau (\mathcal {A})\ge \tau (\mathcal {A^*})$.

We conclude that $\mathcal {A^*}$ is an optimum algorithm for the communication by meeting model.

#### Theorem 3.11

Consider the line search problem in the *communication by meeting* model for two robots with speeds 1 and *v*, respectively. For each $0 < v \le 1$, the algorithm $\mathcal {A^*}$ achieves the optimum competitive ratio:

$$\begin{aligned} \mathsf {CR}(\mathcal {A^*}) = {\left\{ \begin{array}{ll} \frac{1+3v}{1-v} &{}\quad \text {if }\ 0 < v \le \frac{1}{3},\\ 9v &{}\quad \text {if }\ \frac{1}{3} \le v \le 1. \end{array}\right. } \end{aligned}$$

## Wireless Communication

In this model, we have only the *exploration phase* and the *target phase*. Nevertheless, it turns out that the algorithm $\mathcal {A^*}$ presented in Sect. 3.1 is still optimal if the maximum speeds of the two robots are very different: if $v \le \sqrt{17}-4\approx 0.123$. An algorithm $\mathcal {B^*}$ optimal for $v \ge \sqrt{17}-4$ is described in Sect. 4.1. As opposed to $\mathcal {A^*}$, in $\mathcal {B^*}$ both robots participate in the exploration phase. By Corollary 3.9, this is actually necessary to improve upon $\mathcal {A^*}$.

### The Upper Bound

The optimal strategy for two robots traveling at the same speed [5] is very simple: Both robots explore in opposite directions at full speeds. When a robot learns that the other robot has found the target, it changes its direction towards the target.

Let us analyze the performance of this strategy for robots with distinct speeds. The total search time is a sum of three terms: the time for a robot to discover the target, the time for the other robot to go back to the origin and the time for that robot to reach the target. We consider two cases. First, suppose that the faster robot *R* discovers the target at distance *d* from the origin. Then the total search time is $d + d + \frac{1}{v} d = (2+\frac{1}{v})d$. On the other hand, if the slower robot *r* discovers the target, the search time is worse: $\frac{1}{v} d + \frac{1}{v} d + d=(\frac{2}{v}+1)d$.

Intuitively, the faster robot explores too fast and it thus spends too much time going back to the origin. Hence, we limit the progress speed of *R* to $v' \le 1$. When it already knows the target, the faster robot is still allowed to use its full speed equal to 1. Now, the total search times are $\frac{1}{v'}d+\frac{1}{v'}d + \frac{1}{v} d = (\frac{2}{v'}+\frac{1}{v})d$ and $\frac{1}{v} d + \frac{v'}{v}d + d = \frac{1+v'+v}{v}d$, respectively. We choose $v'$ to minimize the maximum of these two quantities. As they are, respectively, a decreasing and an increasing function of $v'$, for the optimal value $v'$ these terms are equal to each other, i.e., $v'$ satisfies $\frac{1+v'+v}{v}=\frac{2}{v'}+\frac{1}{v}$.

**Fig. 5:**
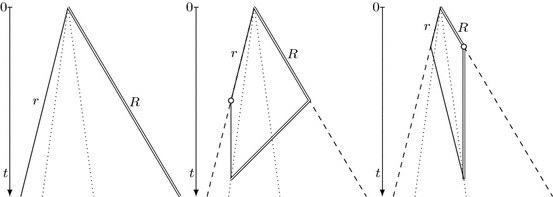
Illustration of algorithm $\mathcal {B^*}$ before target discovery (left), when the target is discovered by *r* (middle), and by *R* (right). The horizontal axis represents the line searched and the vertical axis represents the time. The empty circle denotes the target discovery. Double and single solid lines represent the trajectories of the faster and the slower robot, respectively. Dashed lines correspond to the progress speeds of the two robots and dotted lines to the search time

**Table:** 

**Algorithm**$\mathcal {B^*}$ [for two robots with wireless communication]	
1. Until the target is discovered, the two robots move in opposite directions. Robot *r* moves with its maximal speed *v* and robot *R* with speed $v' = \frac{1}{2}(\sqrt{v^2 + 8v}-v) \le 1$.	
2. When either robot finds the target, it notifies the other one using wireless communication and the other robot moves to the target using its maximal speed.	

The following fact gives the right values of $v'$ and of the search time $\tau ^*$. This lets us complete the description of the algorithm $\mathcal {B^*}$ (see Fig. 5), whose analysis follows immediately from the discussion above.

#### Fact 4.1

For any speed $v\in (0,1]$, let us define $\tau ^*= \tfrac{2+v+\sqrt{v^2 + 8v}}{2v}$ and $v'=\tfrac{\sqrt{v^2 + 8v}-v}{2}$. We have $\tau ^*= \tfrac{1+v+v'}{v}$ and $\tau ^*=\tfrac{1}{v}+\tfrac{2}{v'}$. Moreover, $v'$ is an increasing function of *v*.

#### Proof

By definition of $\tau ^*$ and $v'$,

$$\begin{aligned} \tfrac{1+v+v'}{v}=\tfrac{2+2v+2v'}{2v}=\tfrac{2+2v+\sqrt{v^2 + 8v}-v}{2v}=\tfrac{2+v+\sqrt{v^2 + 8v}}{2v}=\tau ^*. \end{aligned}$$

Similarly,

$$\begin{aligned} \tfrac{1}{v}+\tfrac{2}{v'}=\tfrac{1}{v}+\tfrac{4}{\sqrt{v^2 + 8v}-v}= \tfrac{1}{v}+\tfrac{4(\sqrt{v^2 + 8v}+v)}{(\sqrt{v^2 + 8v}-v)(\sqrt{v^2 + 8v}+v)}= \tfrac{1}{v}+\tfrac{4(\sqrt{v^2 + 8v}+v)}{8v}=\tau ^*. \end{aligned}$$

Finally, note that

$$\begin{aligned} v'=\tfrac{4v}{\sqrt{v^2 + 8v}+v}=\tfrac{4}{\sqrt{1 + 8/v}+1} \end{aligned}$$

indeed is an increasing function of *v*. $\square$

Recall that $t(\mathcal {B^*},p)=|p|(\frac{2}{v'}+\frac{1}{v})$ or $t(\mathcal {B^*},p)=|p|\frac{1+v'+v}{v}$, depending on which robot visits *p* first. Setting $v'$ according to Fact 4.1, in both cases we obtain $t(\mathcal {B^*},p)=\tau ^*|p|$. In other words:

#### Proposition 4.2

For every point $p\in \mathbb {R}$, the algorithm $\mathcal {B^*}$ satisfies $t(\mathcal {B^*},p)=\tfrac{2+v+\sqrt{v^2 + 8v}}{2v} |p|$.

#### Corollary 4.3

For the algorithm $\mathcal {B^*}$, $\mathsf {CR}(\mathcal {B^*})=\tfrac{2+v+\sqrt{v^2+8v}}{2}$.

### The Lower Bound

In this section, we show that for each *v*, the optimum competitive ratio is achieved by either $\mathcal {A^*}$ or $\mathcal {B^*}$. Below, we give an overview of this proof.

**Fig. 6:**
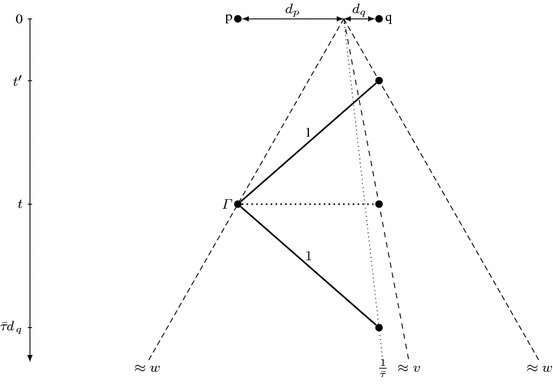
Illustration of notions used in the proof of Lemma 4.4. Here, robot $\varGamma$, while in *p* at time *t*, must be able to reach *q* before the deadline or know that the target is not in *q*. For the latter, one of the robots must have already visited *q*

The argument starts with Lemma 4.4, which is a counterpart of Lemma 3.3. Compared to Lemma 3.3, we allow for the wireless communication, but restrict to the case of two robots and analyze the situation only from the perspective of a robot $\varGamma$ whose progress speed is equal to the overall progress speed *w*. As in the original proof, we fix a point $p=\varGamma (t)$ with $|p|\approx t\cdot w$, choose a point *q* on the opposite side of the origin so that $\varGamma$ cannot reach *q* before the deadline, and conclude that $\varGamma$ must already know at time *t* that the target is not located at *q*. Then, the reasoning becomes different and to proceed, we consider two cases. If *q* has been discovered by $\varGamma$ (which then traveled to *p*), we obtain the familiar upper bound on *T*(*q*), which is then combined with a lower bound following from the limited progress speed. Otherwise, the other robot $\varGamma '$ may have used the wireless communication to notify $\varGamma$, so we can only deduce $T(q)\le t$. However, we observe that the progress speed of $\varGamma '$ is at most *v*, so in this case we get a better lower bound on *T*(*q*); see Fig. 6. Combining all the constraints, after some calculations we obtain the inequality $\tau (\mathcal {B})\ge \min (\frac{1+3w}{w-w^2}, \frac{1+v+w}{v})$.

Our next aim is to prove that a large progress speed excludes improving upon $\mathcal {A^*}$ and $\mathcal {B^*}$. Mimicking the idea behind Corollary 3.5, in Corollary 4.5 we use Lemma 4.4 to show that any algorithm $\mathcal {B}$ with progress speed $w\ge \max (v', \frac{1-v}{1+3v})$ satisfies $\tau (\mathcal {B})\ge \min (\tau (\mathcal {A^*}),\tau (\mathcal {B^*}))$.

**Fig. 7:**
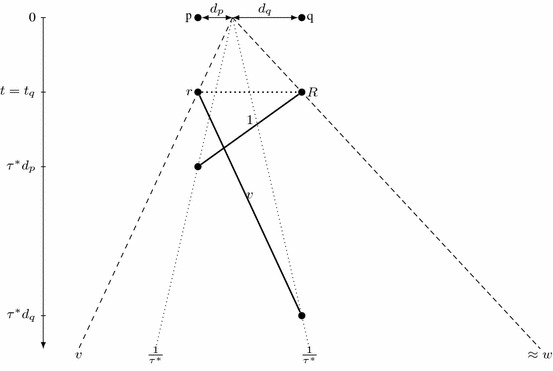
Illustration of notions used in the first part of the proof of Lemma 4.6. The slower robot *r*, while discovering *p* at time *t*, must be able to reach *q* before $\tau ^*d_q$. Similarly, the robot discovering *q* at time $t_q$ must be able to reach *p* before $\tau ^*d_p$

Next, in Lemma 4.6 we prove the optimality of $\mathcal {A^*}$ or $\mathcal {B^*}$ assuming that $w <\max (v', \frac{1-v}{1+3v})$. The proof of this lemma is by far the most intricate reasoning in this paper. We consider a hypothetical algorithm $\mathcal {B}$ with $\tau (\mathcal {B})<\min (\tau (\mathcal {A^*}),\tau (\mathcal {B^*}))$. At the beginning, we note that Corollary 3.9 lets us assume that the slower robot *r* must actively participate in the exploration phase; otherwise, there is no hope to defeat $\mathcal {A^*}$. Thus, we consider a time *t* when the slower robot *r* discovers a point $p=r(t)$, and we define *q* to be the other endpoint of *D*(*t*). If *q* was too close to the origin, then the robot *r* would not have enough time to reach the target located in the vicinity of *q*. On the other hand, the point *q* cannot be too far since the robot (*r* or *R*) discovering *q* cannot exceed the overall progress speed and must be able to reach the target if it was located in the vicinity of *p*; see Fig. 7.

A combination of these constraints lets us conclude that $v'<w < \frac{1-v}{1+3v}$, that *R* must have discovered *q*, and that no robot can visit *q* at time $t'\ge t$ before the vicinity of *p* has been explored. If the robot *r* discovers any point $p'$ before *q* is visited again, then we may replace *t* with $T(p')$ and *p* with $p'$, while preserving the point *q*. Thus, an appropriate choice of *t* (and *p*) lets us guarantee that the slower robot *r* does not discover any further point until *q* has been visited again.

**Fig. 8:**
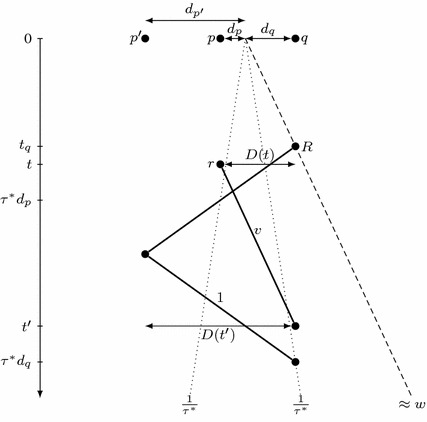
Illustration of notions used in the last part of the proof of Lemma 4.6

Consequently, we examine the earliest time $t'\ge t$ when some robot visits *q*. Note that both endpoints of segment $D(t')$ must have been discovered by the faster robot *R*. We consider two cases depending on the identity of the robot visiting *q*. If this is the faster robot *R*, we bound the length of the segment $D(t')$ relative to the time $t'$. The resulting value contradicts the lower bound of Lemma 3.8 on the discovery speed. Otherwise, we repeat the whole reasoning with $t'$ instead of *t*; see Fig. 8. After sufficiently many iterations, we contradict Lemma 3.8. More precisely, we work with the function $\varDelta (t) := |D(t)|-t\cdot \frac{2v}{v\tau (\mathcal {A^*})-1}$: we show that it decreases each time we change *t*, whereas the lower bound of Lemma 3.8 on the discovery speed implies that it tends to infinity in the limit.

Corollary 4.5 and Lemma 4.6 cover the complementary cases of large and small progress speeds, respectively, so Proposition 4.7 and Theorem 4.8 immediately follow.

In the remainder of this section, we provide formal proofs of the aforementioned claims. First, we relate the progress speed *w* of a strategy $\mathcal {B}$ with its limit search time $\tau (\mathcal {B})$.

#### Lemma 4.4

Let $\mathcal {B}$ be a line search algorithm in the wireless communication model for two robots with speeds 1 and *v*, respectively. If the overall progress speed is *w*, then $\tau (\mathcal {B})\ge \min (\frac{1+3w}{w-w^2}, \frac{1+v+w}{v})$.

#### Proof

Let us choose $\bar{\tau },\varepsilon \in \mathbb {R}$ so that $\bar{\tau }> \tau (\mathcal {B})$ and $\varepsilon > 0$. Then, there exists $d_0>0$ such that $\frac{1}{\bar{\tau }}t(\mathcal {B},p)< |p| < (w+\varepsilon ) T(p)$ for $|p|\ge d_0$. Also, there is a robot $\varGamma$ and arbitrarily large times *t* such that $\frac{|\varGamma (t)|}{t}\ge w-\varepsilon$; we fix one with $t\ge \bar{\tau }d_0$.

Let $p=\varGamma (t)$ and $d_p = |p|$. Also, consider a point *q* at distance $d_q = \frac{t+d_p}{\bar{\tau }- 1}$ from the origin, opposite to *p*, and denote $t'=T(q)$; see Fig. 6. Observe that $d_q \ge \frac{t}{\bar{\tau }}\ge d_0$, so $\frac{1}{\bar{\tau }}t(\mathcal {B},q)< d_q < (w+\varepsilon )t'$.

First, suppose that $t'>t$. Then, the robot $\varGamma$ must be able to reach *q* by the deadline, at $t(\mathcal {B},q)<\bar{\tau }d_q$. Since $\varGamma (t)=p$ and the robot $\varGamma$ cannot exceed the speed limit of 1, we conclude that $t+d_p+d_q < \bar{\tau }d_q$. However, the distance $d_q$ is defined so that $t+d_p+d_q = \bar{\tau }d_q$, a contradiction.

Thus, $t'\le t$. Now, we consider two possibilities for the robot which discovered *q*. If $\varGamma (t')=q$, then $t' \le t - d_p - d_q$ by the speed limit of $\varGamma$. Consequently,

$$\begin{aligned} \tfrac{d_q}{w+\varepsilon }&< t - d_p - d_q,\\ d_q(1+w+\varepsilon )&< (w+\varepsilon )(t-d_p). \end{aligned}$$

By definition of $d_q$, this further yields

$$\begin{aligned} \tfrac{t+d_p}{\bar{\tau }- 1}(1+w+\varepsilon )&< (w+\varepsilon )(t-d_p),\\ (t+d_p)(1+w+\varepsilon )&<(w+\varepsilon )(t-d_p)(\bar{\tau }- 1),\\ d_p(1+w+\varepsilon + (w+\varepsilon )(\bar{\tau }- 1))&<t((w+\varepsilon )(\bar{\tau }-1)-(1+w+\varepsilon )),\\ d_p(1+ (w+\varepsilon )\bar{\tau })&<t((w+\varepsilon )(\bar{\tau }-2)-1),\\ \tfrac{d_p}{t}&<\tfrac{(w+\varepsilon )(\bar{\tau }-2)-1}{1+ (w+\varepsilon )\bar{\tau }}. \end{aligned}$$

Otherwise, either $\varGamma = R$ and *r* discovered *q*, or $\varGamma = r$ in which case $w \le v$. In both sub-cases, we have $d_q < T(q)(v+\varepsilon )=t'(v+\varepsilon )\le t(v+\varepsilon )$. Combining this inequality with the definition of $d_q$, we obtain

$$\begin{aligned} \tfrac{t+d_p}{\bar{\tau }- 1}&<t(v+\varepsilon ),\\ t+d_p&<t(v+\varepsilon )(\bar{\tau }-1),\\ d_p&< t(v+\varepsilon )(\bar{\tau }-1)-t,\\ \tfrac{d_p}{t}&< (v+\varepsilon )(\bar{\tau }-1)-1. \end{aligned}$$

Summing up, the two cases yield

$$\begin{aligned} \tfrac{d_p}{t}\le \max \left( (v+\varepsilon )(\bar{\tau }-1)-1, \tfrac{(w+\varepsilon )(\bar{\tau }-2)-1}{(w+\varepsilon )\bar{\tau }+1}\right) . \end{aligned}$$

However, recall that the time *t* was chosen so that $\frac{d_p}{t}\ge w-\varepsilon$. Therefore,

$$\begin{aligned} w-\varepsilon \le \max \left( (v+\varepsilon )(\bar{\tau }-1)-1, \tfrac{(w+\varepsilon )(\bar{\tau }-2)-1}{(w+\varepsilon )\bar{\tau }+1}\right) . \end{aligned}$$

As $\varepsilon > 0$ can be chosen arbitrarily close to 0, we conclude thati.e., $\bar{\tau }\ge \min (\frac{1+3w}{w-w^2}, \frac{1+v+w}{v})$ for each $\bar{\tau }> \tau (\mathcal {B})$. Consequently,

$$\begin{aligned} \tau (\mathcal {B})\ge \min \left( \tfrac{1+3w}{w-w^2}, \tfrac{1+v+w}{v}\right) , \end{aligned}$$

as claimed. $$\square $$

We apply Lemma 4.4 to derive a tight lower bound on $\tau (\mathcal {B})$ provided that the overall progress speed *w* is sufficiently large.

#### Corollary 4.5

Let $\mathcal {B}$ be a line search algorithm in the wireless communication model for two robots with speeds 1 and *v*, respectively. If the overall progress speed is $w \ge \max (v', \tfrac{1-v}{1+3v})$, then $\tau (\mathcal {B})\ge \min (\tau (\mathcal {A^*}),\tau (\mathcal {B^*}))$.

#### Proof

Let us recall that the function $f(v)=\frac{1+3v}{v-v^2}$ is decreasing for $0<v\le \frac{1}{3}$ and increasing for $\frac{1}{3} \le v < 1$. For $v\le \tfrac{1}{3}$, we have $\frac{1-v}{1+3v}\ge \frac{1}{3}$. Hence, due to $w \ge \max (v',\frac{1-v}{1+3v})\ge \frac{1}{3}$, Lemma 4.4 yields

$$\begin{aligned} \tau (\mathcal {B})\ge \min \left( f(w),\tfrac{1+v+w}{v}\right) \ge \min \left( f(\tfrac{1-v}{1+3v}), \tfrac{1+v+v'}{v}\right) \ge \min (\tau (\mathcal {A^*}),\tau (\mathcal {B^*})). \end{aligned}$$

On the other hand, for $v \ge \frac{1}{3}$ Lemma 4.4 implies

$$\begin{aligned} \tau (\mathcal {B})\ge \min \left( f(w),\tfrac{1+v+w}{v}\right) \ge \min \left( f(\tfrac{1}{3}), \tfrac{1+v+v'}{v}\right) \ge \min \left( \tau (\mathcal {A^*}),\tau (\mathcal {B^*})\right) . \end{aligned}$$

In both cases we derived the claimed inequality. $\square$

In the next lemma, we prove the same lower bound on $\tau (\mathcal {B})$ for the complementary case of small progress speed.

#### Lemma 4.6

Let $\mathcal {B}$ be a line search algorithm in the wireless communication model for two robots with speeds 1 and *v*, respectively. If the overall progress speed *w* satisfies $w<\max (v',\frac{1-v}{1+3v})$, then $\tau (\mathcal {B})\ge \min (\tau (\mathcal {A^*}),\tau (\mathcal {B^*}))$.

#### Proof

For a proof by contradiction, suppose that $\tau (\mathcal {B})< \tau ^*$, where $\tau ^*=\min (\tau (\mathcal {A^*}),\tau (\mathcal {B^*}))$. Consequently, also due to the upper bound on *w*, there exists $d_0>0$ such that $\frac{1}{\tau ^*}t(\mathcal {B},p)< |p|< T(p)\max (v',\frac{1-v}{1+3v})$ for $|p|\ge d_0$.

By Corollary 3.9, $\tau (\mathcal {B})< \tau (\mathcal {A^*})$ implies that the set of points discovered by the slower robot *r* is unbounded. Thus, the set $T:= \{t : T(r(t))=t\}$ of times when the slower robot *r* discovers some point is also unbounded.

By Lemma 3.8, the discovery speed is at least $v_d \ge \tfrac{2v}{v\tau (\mathcal {B})-1}>\tfrac{2v}{v \tau ^*- 1}$, i.e., $\liminf _{t\rightarrow \infty } \tfrac{|D(t)|}{t} > \tfrac{2v}{v\tau ^*-1}$. Consequently, the function

$$\begin{aligned} \varDelta (t) := |D(t)|-t\tfrac{2v}{v\tau ^*-1} \end{aligned}$$

satisfies $$\lim _{t\rightarrow \infty } \varDelta (t) = \infty $$.

Hence, for each threshold $\delta$, the set $T_{\delta } := \{t \in T : \varDelta (t)\le \delta \}$ is bounded. Due to the structure of the set *T*, this implies $t_\delta := \sup T_{\delta } \in T_{\delta }$. As a result, we obtain arbitrarily large times $t = t_{\delta }\in T$ such that $\varDelta (t) < \varDelta (t')$ for each $t'\in T$ with $t'>t$;

Let us take $t = t_{\delta }$ for some $\delta \ge 0$ such that $t \ge \tau ^*d_0$. Moreover, let $p=r(t)$ and let *q* the other endpoint of the segment *D*(*t*). Let us denote $d_p=|p|$ and $d_q = |q|$; see Fig. 7. Observe that $d_p,d_q\ge d_0$ because $t \ge \tau ^*d_0$ yields $[-d_0,d_0]\subseteq D(t)$. (Otherwise, the target at distance $d_0$ from the origin would not be discovered prior to the deadline, which does not exceed $\bar{\tau }d_0$.)

At time *t*, there are undiscovered points arbitrarily close to *q*, so the robot *r* must be able to reach *q* before $\tau ^*d_q$. Consequently, $t + \frac{1}{v}(d_p+d_q) < \tau ^*d_q$, i.e., $d_q > \frac{tv+d_p}{v\tau ^*-1}$. By the speed limit on *r*, we have $d_p \le tv$, and therefore

$$\begin{aligned} d_q > \tfrac{tv+d_p}{v\tau ^*-1}\ge \tfrac{2d_p}{v\tau ^*-1}\ge \tfrac{2d_p}{v\tau (\mathcal {B^*})-1}\ge \tfrac{2d_p}{v\big (\tfrac{1}{v}+\tfrac{2}{v'}\big )-1}=\tfrac{v'}{v}d_p. \end{aligned}$$

By time *t* and, in general, as long as there are undiscovered points arbitrarily close to *p*, any robot $\varGamma$ visiting *q* must be able to reach *p* before $\tau ^*d_p$. Such a visit of *q* is possible only at times $t'$ which satisfy $t' + (d_p+d_q) < \tau ^*d_p$. Hence,

$$\begin{aligned} \tfrac{t'}{d_p}< \tau ^*-1 - \tfrac{d_q}{d_p} < \tau ^*-1 -\tfrac{v'}{v} \le \tau (\mathcal {B^*})-1 -\tfrac{v'}{v} = \tfrac{1+v+v'}{v}-1-\tfrac{v'}{v}=\tfrac{1}{v}, \end{aligned}$$

which yields $t' < \tfrac{d_p}{v} \le t$. In other words, after time *t*, no robot can visit point *q* again until the neighborhood of *p* has been discovered.

Moreover, the discovery time $t_q := T(q)$ satisfies

$$\begin{aligned}&\tfrac{t_q}{d_q}< (\tau ^*-1)\tfrac{d_p}{d_q} - 1 < (\tau ^*-1)\tfrac{v}{v'} -1 \le (\tau (\mathcal {B^*})-1)\tfrac{v}{v'} -1\\&\qquad = (\tfrac{1+v+v'}{v}-1)\tfrac{v}{v'}-1=\tfrac{1}{v'}. \end{aligned}$$

Due to $v'\ge v$, this means that *q* has been discovered by the faster robot *R*. We also have $d_q < t_q \max (v', \tfrac{1-v}{1+3v})$, which lets us conclude that

$$\begin{aligned} t_q v'< d_q < t_q \tfrac{1-v}{1+3v}. \end{aligned}$$

Next, we shall prove that, after time *t*, the slower robot *r* cannot discover any new point until *q* is visited again. For a proof by contradiction, suppose that it discovers point $p'$ (at distance $|p'|=d_{p'}$ from the origin, on the same side as *p*) at time $t'>t$, $t'\in T$, and *q* is not visited between time *t* and $t'$. Note that $t'\ge t+\frac{1}{v}(d_{p'}-d_p)$, $D(t')=\overline{p'q}$, and $D(t)=\overline{pq}$. By the choice of $t\in T$, we have $\varDelta (t) < \varDelta (t')$, i.e.,

$$\begin{aligned} |D(t)|-t\tfrac{2v}{v \tau ^*- 1} < |D(t')|-t'\tfrac{2v}{v \tau ^*- 1}\le |D(t')| - t\tfrac{2v}{v \tau ^*- 1} -(d_{p'}-d_p)\tfrac{2}{v\tau ^*- 1}, \end{aligned}$$

and therefore

$$\begin{aligned} d_{p'}-d_p =|D(t')|-|D(t)| > \tfrac{2(d_{p'}-d_p)}{v\tau ^*- 1} \ge \tfrac{2(d_{p'}-d_p)}{v\tau (\mathcal {B^*}) - 1}=\tfrac{v'(d_{p'}-d_p)}{v}\ge d_{p'}-d_p, \end{aligned}$$

a contradiction. Consequently, the robot *r* cannot discover any point before *q* is visited again after time *t*. As we have already proved, the latter may happen only after *R* visits *p* (and discovers its neighborhood).

Let $t'>t$ be the time when *q* is visited for the first time after *t*. Let $p'$ be the furthest point opposite to *q* discovered (by *R*) prior to time $t'$, and let $d_{p'}=|p'|$; see Fig. 8. There are undiscovered points arbitrarily close to *q*, so $t' < \tau ^*d_q \le \tau (\mathcal {A^*})d_q \le \frac{1+3v}{v-v^2}d_q\le \frac{t_q}{v}$.

Now, we consider two cases depending on which robot first visits *q* at time $t'$. First, suppose that it is the faster robot *R*. By the speed limit, we have $t' \ge t_q + 2(d_{p'}+d_q)$. Since $t'>t = t_\delta$ for $\delta \ge 0$, we have $\varDelta (t)>0$, i.e.,

$$\begin{aligned} \tfrac{|D(t')|}{t'}> \tfrac{2v}{v\tau ^*- 1} \ge \tfrac{2v}{v\tau (\mathcal {A^*}) - 1}\ge \tfrac{2v}{\tfrac{1+3v}{1-v} - 1}=\tfrac{1-v}{2}. \end{aligned}$$

On the other hand,

$$\begin{aligned} \tfrac{|D(t')|}{t'}=\tfrac{d_{p'}+d_q}{t'} \le \tfrac{t'-t_q}{2t'} = \tfrac{1}{2}-\tfrac{t_q}{2t'}<\tfrac{1}{2}-\tfrac{v}{2}=\tfrac{1-v}{2}. \end{aligned}$$

These two inequalities contradict each other.

Consequently, it must be the slower robot *r* which visits *q* at time $t'\ge t+\frac{1}{v}(d_p+d_q)$. Hence, $t'\in T$ and, by definition of $t\in T$, this yields $\varDelta (t)<\varDelta (t')$, i.e.,

$$\begin{aligned} |D(t)|-t\tfrac{2v}{v \tau ^*- 1} < |D(t')|-t'\tfrac{2v}{v \tau ^*- 1}\le |D(t')| - t\tfrac{2v}{v \tau ^*- 1}-(d_p+d_q)\tfrac{2}{v\tau ^*-1}. \end{aligned}$$

Consequently,

$$\begin{aligned} d_{p'}-d_p= & {} |D(t')|-|D(t)| \ge (d_p+d_q)\tfrac{2}{v\tau ^*-1} \ge (d_p+d_q)\tfrac{2}{v\tau (\mathcal {A^*})-1}\\\ge & {} (d_p+d_q)\tfrac{1-v}{2v}, \end{aligned}$$

and therefore $d_p \tfrac{1+v}{2v} + d_q \tfrac{1-v}{2v} \le d_{p'}$. However, since *R* must be able to reach *q* before $\tau ^*d_q$, we have

$$\begin{aligned} t_q + 2(d_q+d_{p'})< \tau ^*d_q \le \tau (\mathcal {A^*})d_q\le \tfrac{1+3v}{v-v^2}d_q, \end{aligned}$$

and thus

$$\begin{aligned} d_p' < \tfrac{\frac{1+3v}{v-v^2}d_q-t_q}{2}-d_q=\tfrac{1+3v}{2(v-v^2)}d_q-d_q-\tfrac{t_q}{2}. \end{aligned}$$

Moreover, *R* must be able to reach *p* before $\tau ^*d_p$, so

$$\begin{aligned} t_q + (d_q+d_p)< \tau ^*d_p \le \tau (\mathcal {A^*})d_p \le \tfrac{1+3v}{v-v^2}d_p, \end{aligned}$$

and therefore

$$\begin{aligned} d_p \ge \tfrac{t_q+d_q}{\frac{1+3v}{v-v^2} - 1}=\tfrac{(t_q+d_q)(v-v^2)}{(1+v)^2}. \end{aligned}$$

Hence,

$$\begin{aligned} \tfrac{(t_q+d_q)(v-v^2)}{(1+v)^2} \tfrac{1+v}{2v} + d_q \tfrac{1-v}{2v}&< \tfrac{1+3v}{2(v-v^2)}d_q-d_q-\tfrac{t_q}{2},\\ \tfrac{(t_q+d_q)(1-v)}{2(1+v)}+ d_q \tfrac{1-v}{2v}&< \tfrac{1+3v}{2(v-v^2)}d_q-d_q-\tfrac{t_q}{2},\\ t_q \left( \tfrac{1}{2}+\tfrac{1-v}{2(1+v)}\right)&< d_q\left( \tfrac{1+3v}{2(v-v^2)}-1- \tfrac{1-v}{2v}-\tfrac{1-v}{2(1+v)}\right) ,\\ t_q \tfrac{1+v+1-v}{2(1+v)}&< d_q\left( \tfrac{1+3v}{2v(1-v)}-\tfrac{1+v}{2v}-\tfrac{1-v}{2(1+v)}\right) ,\\ t_q \tfrac{2}{2(1+v)}&< d_q\left( \tfrac{1+3v}{2v(1-v)}-\tfrac{(1+v)^2+v(1-v)}{2v(1+v)}\right) ,\\ t_q \tfrac{1}{1+v}&< d_q\left( \tfrac{1+3v}{2v(1-v)}-\tfrac{1+2v+v^2+v-v^2}{2v(1+v)}\right) ,\\ t_q&< d_q\left( \tfrac{(1+3v)(1+v)}{2v(1-v)}-\tfrac{1+3v}{2v}\right) ,\\ t_q&< d_q\tfrac{(1+3v)((1+v)-(1-v))}{2v(1-v)},\\ t_q&< d_q\tfrac{1+3v}{1-v}. \end{aligned}$$

However, this contradicts $d_q < t_q\tfrac{1-v}{1+3v}$. $\square$

Corollary 4.5 and Lemma 4.6 let us conclude the tight lower bound.

#### Proposition 4.7

Any algorithm $\mathcal {B}$ in the wireless communication model for two robots with speeds 1 and *v*, respectively, satisfies $\tau (\mathcal {B})\ge \min (\tau (\mathcal {A^*}),\tau (\mathcal {B^*}))$.

We conclude that for each *v*, either $\mathcal {A^*}$ or $\mathcal {B^*}$ is an optimum algorithm for the wireless communication model. Simple calculations show that $\mathcal {A^*}$ is optimal for $v\le \sqrt{17}-4$, whereas $\mathcal {B^*}$ is optimal for $v \ge \sqrt{17}-4$.

#### Theorem 4.8

Consider the line search problem in the wireless communication model for two robots with speeds 1 and *v*, respectively. For each $0 < v \le 1$, either the algorithm $\mathcal {A^*}$ or $\mathcal {B^*}$ achieves the optimum competitive ratio:

$$\begin{aligned} \min (\mathsf {CR}(\mathcal {A^*}),\mathsf {CR}(\mathcal {B^*})) = {\left\{ \begin{array}{ll} \mathsf {CR}(\mathcal {A^*})=\frac{1+3v}{1-v} &{}\quad \text {if }\ 0 < v \le \sqrt{17}-4,\\ \mathsf {CR}(\mathcal {B^*})= \frac{2+v+\sqrt{v^2+8v}}{2} &{}\quad \text {if }\ \sqrt{17}-4 \le v \le 1. \end{array}\right. } \end{aligned}$$
